# Vitreous Hyper-Reflective Dots in Optical Coherence Tomography and Cystoid Macular Edema after Uneventful Phacoemulsification Surgery

**DOI:** 10.1371/journal.pone.0095066

**Published:** 2014-04-15

**Authors:** Jong-Hyun Oh, Roy S. Chuck, Jae Rock Do, Choul Yong Park

**Affiliations:** 1 Department of Ophthalmology, Dongguk University Ilsan Hospital, Goyang, South Korea; 2 Department of Ophthalmology and Visual Sciences, Montefiore Medical Center, Albert Einstein College of Medicine, Bronx, New York, United States of America; Massachusetts Eye & Ear Infirmary, Harvard Medical School, United States of America

## Abstract

**Purpose:**

To report the observation of hyper-reflective dots in the vitreous cavity using spectral domain optical coherence tomography (SD-OCT) after uneventful phacoemulsification cataract surgery and to investigate their association with cystoid macular edema (CME).

**Materials and Methods:**

Medical records of consecutive Asian patients who had no preoperative retinopathy and underwent uneventful phacoemulsification cataract surgery from March 2012 through February 2013 were reviewed. SD-OCTs were performed before, 1 week, and 1 month after surgery. The number of vitreous hyper-reflective dots (VHDs) was counted in 5 OCT images of high-definition 5-line raster scans. The development of CME was assessed using postoperative 1-month OCT.

**Results:**

In 74 eyes of 74 patients, all of three SD-OCTs with a signal to noise ratio of 0.6 or more were available and were analyzed in this study. In preoperative OCT, the VHD was observed in 2 (2.7%) of 74 eyes; one eye had 1 VHD and the other eye had 2 VHDs. In 72 eyes with no preoperative VHD, VHDs were observed in 40 (55.6%) eyes at 1 week after the surgery. In the multivariate analysis, the number of VHDs measured at 1 week after the surgery was significantly associated with CME development at 1 month after the surgery (odds ratio = 1.93, 95% confidence interval = 1.15 to 3.24, *P* = 0.012).

**Conclusions:**

VHDs were frequently observed in OCT after uneventful phacoemulsification cataract surgery. VHDs observed at 1 week after the surgery may be a risk factor for the development of pseudophakic CME. Further studies are needed to identify the source of the VHDs.

## Introduction

Pseudophakic cystoid macular edema (CME) is a common cause of unexpected visual loss after cataract surgery. The exact pathogenesis of CME is unclear; however, increased vascular permeability by inflammatory mediators may play a critical role. [1] Various preoperative and postoperative factors are proposed to increase CME after cataract surgery; iris trauma or posterior capsule rupture, vitreous loss, diabetic retinopathy, retinal vein occlusion, epiretinal membrane, or uveitis are some of those factors. [2]–[6] It is not uncommon; however, to encounter CME in otherwise healthy eyes after uneventful phacoemulsification surgery [7], [8].

**Figure 1:**
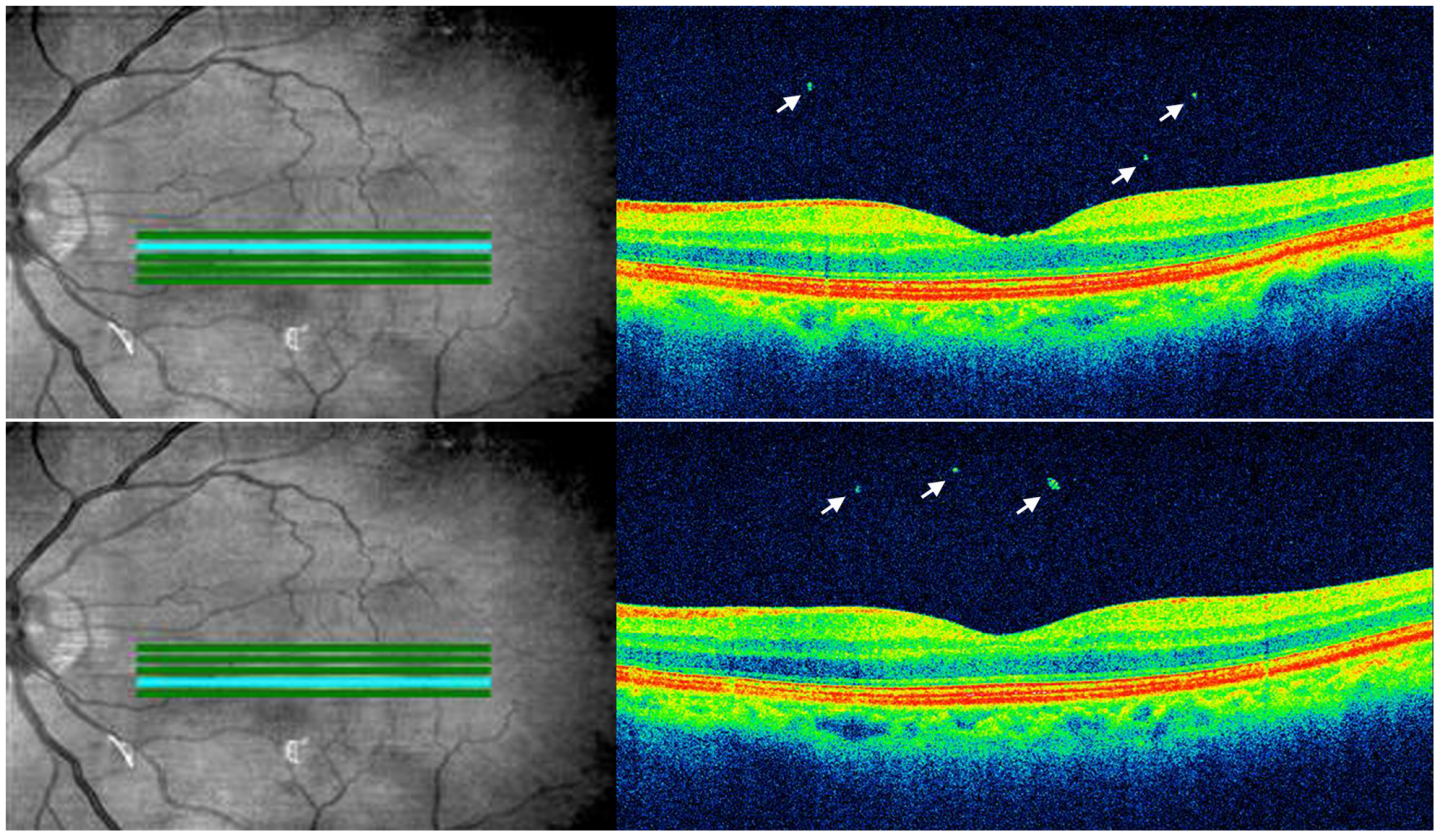
OCT images of an eye 1 month after uneventful phacoemulsification cataract surgery. In the vitreous cavity, hyper-reflective dots (arrows) are observed on OCT images of a high-definition 5-line raster scan images.

Optical coherence tomography (OCT) can obtain high-resolution cross-sectional images of various parts of the eye. It was initially utilized to evaluate the retina and the optic nerve head, but with the advance of the technology, OCT is now used to evaluate additionally other parts of the eye including cornea, tear film, choroid and vitreous body. [9]–[14] Using high-resolution, spectral domain (SD) OCT, we observed some hyper-reflective dots in the vitreous cavity after uneventful phacoemulsification cataract surgery (Figure 1).

In the present study, we analyzed the incidence of these vitreous hyper-reflective dots (VHDs) and their association with the development of CME after uneventful phacoemulsification surgery.

## Materials and Methods

This study was designed as a retrospective case series. Written consent was given by the patients for their information to be stored in the hospital database and used for research. Institutional review board approval was obtained from the Dongguk University Ilsan Hospital, Goyang, South Korea. All research adhered to the tenets of the Declaration of Helsinki.

### Patients and Data Collection

We retrospectively reviewed the medical records of consecutive patients who underwent phacoemulsification cataract surgery by a single surgeon (C.Y.P.) from March 2012 through February 2013 at Dongguk University Ilsan Hospital, Goyang, South Korea. Patients who underwent OCT examinations before, 1 week, and 1 month after surgery were included in this study. If data from both eyes were available, only the first operative eyes were included. Eyes with retinopathy including diabetic retinopathy, retinal vein occlusion, epiretinal membrane, and age-related macular degeneration, were excluded from the study. Cases with intraoperative complications, such as posterior capsule rupture, were also excluded.

Data including age, sex, history of diabetes, laterality of the eye, best-corrected Snellen visual acuity (BCVA), and biometric values were obtained. SD-OCTs with a signal to noise ratio of 0.6 or more were assessed for the study.

### Patient Managements and Surgical Technique

Before cataract surgery, patients were subjected to ophthalmic examinations including BCVA measurements, biomicroscopic examination, applanation tonometry, indirect ophthalmoscopy, SD-OCT, and when needed, fluorescein angiography. In addition, partial coherence interferometry or ultrasound biometry, and Scheimpflug imaging were performed to obtain ocular biometric values. Postoperative medication included topical 1% prednisolone applied as 1 drop 4 times per day for 4 weeks, and 0.5% levofloxacin applied 4 times per day for 4 weeks. In addition to BCVA measurements, patients underwent SD-OCT 1 week and 1 month after surgery. Fundus examination was routinely performed 1 month after surgery.

The same surgical techniques were used by a single surgeon (C.Y.P.) in all cases. Under topical anesthesia, a corneal paracentesis was made with a 1-mm diamond knife and then a temporal clear corneal incision was made with a 2.8-mm diamond knife. After the anterior chamber was filled with 4% chondroitin sulfate–3% sodium hyaluronate (Viscoat, Alcon Laboratories, Fort Worth, TX), a continuous curvilinear capsulorhexis of 5 to 5.5 mm was created with a forceps followed by hydrodissection with balanced salt solution using a hydration cannula. Phacoemulsification was performed with the Stellaris phacoemulsification machine (Bausch and Lomb, Rochester, NY). A phaco chop technique was used with the following parameters: 400 mmHg vacuum, 20% initial power, and 110 cm bottle height. Phacoemulsification power was adjusted depending on the density of the lens nucleus. Automated aspiration of the remaining soft lens matter was set at 500 mmHg vacuum and 110 cm bottle height and then followed by capsule polish with a 15 mmHg vacuum and 80 cm bottle height. A single-piece hydrophobic acrylic intraocular lens (Tecnis, Abbott Medical Optics, Inc. Santa Ana, CA) was implanted in the capsular bag with an injector system and residual viscoelastic material was removed from the anterior chamber. After the hydro-sealing technique with a balanced salt solution was applied to the temporal corneal incision, the anterior chamber was reformed with balanced salt solution.

### Optical Coherence Tomography

SD-OCT was performed using the Cirrus™ HD-OCT (Model 4000, software version 6.0, Carl Zeiss Meditec, Dublin, CA) using a wavelength of 840 nm and an axial resolution of 5 µm. All SD-OCT included a macular cube scan and a high-definition 5-line raster scan. The 512×128 macular cube scan produced 128 horizontal scan lines, each comprising 512 A-scans per line, of a 6 mm×6 mm area. Central subfield retinal thickness (CRT) was assessed at the macular center (1 mm diameter) using a topographic map of the macular cube scan. The high-definition 5-line raster scan, which is less affected by ocular movement due to the short scanning time, produced 5 horizontal scan lines that were 6 mm long separated by 250 µm; each scan line comprised 4096 A scans.

VHDs were identified in 5 color images of the high-definition 5-line raster scan. A VHD was defined as a hyper-reflective round- or rod-shaped dot in the vitreous cavity with a vertical length of a least 20 µm (Figure 1). The number of VHDs was counted in each image and summed twice by each of two independent observers (J.-H.O., J.R.D.). Vertical and horizontal lengths of each VHD were measured respectively with OCT software. The diameter of VHD was calculated from the average of vertical and horizontal lengths. CME was defined based on OCT as increased CRT of at least 300 µm or the presence of intraretinal cystoid spaces, defined as relative hypo-reflective areas with a surrounding hyper-reflective boundary and with the shortest diameter of at least 50 µm in macular cube scan images [2], [15].

### Ocular Biometric Values

Keratometric values, anterior chamber depth (ACD), and anterior chamber angle were measured with Scheimpflug imaging (Pentacam, version 1.17r24, Oculus Inc., Wetzlar, Germany). The ACD was measured from the endothelium to the anterior surface of the lens. Axial length was measured with partial coherence interferometry (IOLMaster, version 5.0, Carl Zeiss Meditec) or when needed, ultrasound biometry (Compact II, Quantel Medical, Cedex, France).

### Statistical Analyses

All data were analyzed using SPSS software version 20.0 (SPSS Inc., Chicago, IL, USA). Mean, standard deviation, median, and proportion were used, as appropriate, as descriptive statistics. The Kolmogorov-Smirnov test was used to verify the normality of the distribution of continuous variables. Intra-observer reproducibilities for counting the number of VHDs were assessed using an intraclass correlation coefficient (ICC). Inter-observer ICC also measured using the number of VHDs first counted by each of two independent observers. The number of VHDs first counted by J.-H.O. was used for other analyses. For comparison between groups, the chi-square analysis or Fisher’s exact test was used for categorical variables and the t-test or Mann-Whitney U test was used for continuous variables. The changes in CRT or BCVA were analyzed using a paired t-test. To identify factors for predicting CME development, multivariate logistic regression analyses were used. Odds ratios (ORs) and 95% confidence intervals (CIs) were calculated. The correlation between the number of VHD at 1week after surgery and CME development was assessed with Chi-square test for trend. All statistics were two-tailed, and a *P* value less than 0.05 was considered significant.

## Results

One hundred ninety-six eyes of 147 Asian patients who underwent phacoemulsification cataract surgery were retrospectively reviewed. Seven eyes with diabetic retinopathy, 4 eyes with retinal vein occlusion, 4 eyes with epiretinal membrane, 2 eyes with age-related macular degeneration, and 3 eyes with an intraoperative complication of posterior capsule rupture were excluded from the study. No eyes had a history of uveitis or previous intraocular surgery such as filtering surgery or pars plana vitrectomy. Of the remaining 176 eyes, 100 eyes of 74 patients were performed all of three SD-OCTs with a signal to noise ratio of 0.6 or more before, 1 week and 1 month after surgery. Seventy-four eyes of 74 patients (33 men and 41 women) were analyzed for the study. Mean age was 68.5±9.85 years (range, 47–89), and there were 37 (50%) right eyes and 37 (50%) left eyes. In all 74 eyes, there were no serious postoperative complications, including retinal tear, retinal detachment, or endophthalmitis.

### Vitreous Hyper-reflective Dots in OCT

Intra-observer ICCs for counting the number of VHDs were 0.995 [95% confidence interval (CI) = 0.993 to 0.997, *P*<0.001] and 0.991 (95% CI = 0.986 to 0.994, *P*<0.001) respectively, and inter-observer ICC was 0.989 (95% CI = 0.982 to 0.993, *P*<0.001).

In preoperative OCT, 2 (2.7%) of 74 eyes had the VHD; one eye had one VHD and the other eye had two VHDs. The median diameter of preoperative VHDs was 32 µm (range, 25–32). The remaining 72 eyes had no VHDs detected preoperatively. We performed further analysis using these 72 eyes. At 1 week after the surgery, VHDs were observed in 40 (55.6%) eyes; 18 (25.0%) eyes had one VHD, 8 (11.1%) eyes had two VHDs, and the other 14 (19.4%) eyes had at least three VHDs. Median diameter of VHDs was measured as 40 µm (range, 20–72). At 1 month after the surgery, OCTs of 72 eyes revealed VHDs in 41 (56.9%) eyes; 12 (16.7%) eyes had one VHD, 8 (11.1%) eyes had two VHDs, and the other 21 (29.2%) eyes had at least three VHDs. Median diameter of VHDs was measured as 40 µm (range, 20–78).

### Factors Associated with the Presence of VHDs at 1 Week after the Surgery

Seventy two eyes with no preoperative VHD were divided into two groups according to the existence of VHD at 1 week after surgery. The characteristics of the two groups are shown in Table 1. Mean axial length in eyes with VHDs was significantly shorter than eyes without VHDs (*P* = 0.039).

**Table 1:** Characteristics of eyes with or without VHDs at 1

		Eyes		
		without VHD	With VHD(%)*	
Factor		(n = 32)	(n = 40)	*P* value
Age, years		67.2±10.74	69.6±9.16	0.314
Sex, n				
	male	14	18 (56.3%)	0.916
	female	18	22 (55.0%)	
History of diabetes, n				
	no	27	34 (55.7%)	0.999^†^
	yes	5	6 (54.5%)	
Laterality, n				
	OD	13	23 (63.9%)	0.155
	OS	19	17 (47.2%)	
Preoperative BCVA, logMAR		0.33±0.273	0.30±0.258	0.565
Snellen equivalent		20/43	20/40	
Biometric values				
Mean keratometric value, D		42.6±1.21	43.0±1.60	0.278
Anterior chamber depth, mm		2.71±0.439	2.59±0.340	0.172
Anterior chamber angle, degrees		34.2±5.92	31.8±6.44	0.121
Axial length, mm		24.2±1.14	23.6±1.04	0.039
Preoperative CRT, µm		243±21.6	242±22.3	0.727
Postoperative 1-month BCVA, logMAR		0.11±0.137	0.10±0.175	0.766
Snellen equivalent		20/26	20/25	
Postoperative 1-month CRT, µm		254±24.9	259±33.0	0.476

VHD = vitreous hyper-reflective dot; BCVA = best-corrected visual acuity; logMAR = the logarithm of the minimum angle of resolution; D = diopters; CRT = central subfield retinal thickness.

Results are expressed a mean ± standard deviation.

*Incidence of VHDs in total cases of stated condition.

*P* values were calculated with the chi-square analysis or Fisher exact test^†^ for categorical variables and t-test for continuous variables.

### The Change of CRT and BCVA in Eyes with or without VHDs

In 72 eyes with no preoperative VHD, mean CRT increased from 242±21.9 µm to 257±29.6 µm and mean BCVA improved from 0.31±0.264 logMAR to 0.10±0.158 logMAR at 1 month after surgery (*P*<0.001 and *P*<0.001 respectively). Mean postoperative CRT and BCVA showed no significant difference between eyes with or without VHDs at 1month after surgery (*P = *0.476 and *P* = 0.766 respectively, Table 1).

### VHDs and Cystoid Macular Edema

**Figure 2:**
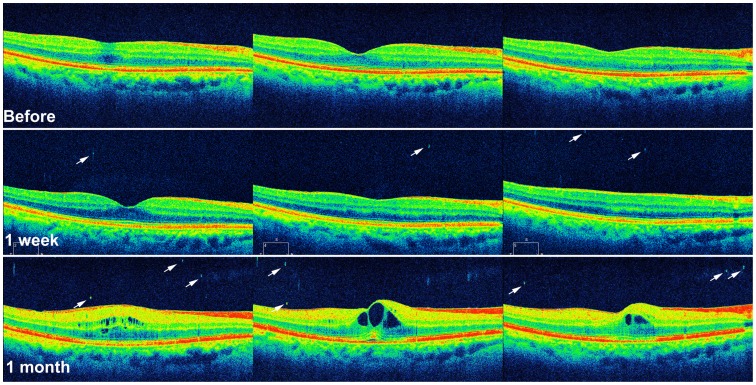
Serial OCT images of an eye with cystoid macular edema at 1 month after uneventful phacoemulsification cataract surgery. VHDs (arrows) are observed at 1 week and 1 month after the surgery in OCT and intraretinal cystoid spaces are observed in OCT at 1 month after the surgery.

OCT-proven CME developed in 10 (13.9%) of 72 eyes at 1 month after surgery. On OCT, 4 eyes had both intraretinal cystoid spaces and increased CRT of at least 300 µm, 5 eyes showed only intraretinal cystoid spaces, and the other one eye showed only increased CRT (317 µm). The characteristics of the groups with or without CME are shown in Table 2. Age, sex, history of diabetics, laterality, preoperative BCVA, and preoperative biometric values did not differ between two groups with or without CME. The mean postoperative CRT measured at 1 month after surgery was significantly thicker in the group with CME than that in the group without CME (*P = *0.002); however, the mean postoperative BCVA did not differ between two groups (*P = *0.811). The number of VHDs at 1 week and 1 month postoperatively was significantly greater in the group with CME than in the group without CME (*P = *0.025 and *P* = 0.004, respectively). In the multivariate analysis, age (OR = 1.14, 95% CI = 1.01 to 1.28, *P* = 0.032) and the number of postoperative 1-week VHDs (OR = 1.93, 95% CI = 1.15 to 3.24, *P* = 0.012) were significantly associated with CME development at 1 month after surgery (Table 3 and Figure 2). The number of postoperative 1-week VHDs was significantly correlated with CME development (*P* = 0.001, Table 4).

**Table 2:** Characteristics of groups with or without CME in OCT 1 month after surgery.

			CME development		
			no	yes (%)*	
Factor			(n = 62)	(n = 10)	*P* value
Age, years			67.9±10.05	72.4±8.25	0.189
Sex, n					
		male	26	6 (18.8%)	0.317
		female	37	4 (9.8%)	
History of diabetes, n					
		no	53	9 (14.5%)	0.999
		Yes	10	1 (9.1%)	
Laterality, n					
		OD	32	5 (13.5%)	0.999
		OS	31	5 (13.9%)	
Preoperative BCVA, logMAR			0.31±0.272	0.35±0.216	0.337
Snellen equivalent			20/41	20/45	
Biometric values					
	Mean keratometric value, D		42.8±1.38	42.9±1.88	0.968
	Anterior chamber depth, mm		2.65±0.394	2.58±0.375	0.563
	Anterior chamber angle, degrees		33.1±6.50	31.2±4.61	0.449
	Axial length, mm		23.9±1.07	23.8±1.41	0.654
	Preoperative CRT, µm		241±20.0	249±31.6	0.479
Postoperative 1-month BCVA, logMAR			0.11±0.160	0.09±0.151	0.811
	Snellen equivalent		20/26	20/25	
Postoperative 1-month CRT, µm			251±21.1	296±44.6	0.002
Median number of 1-week VHDs, n (range)			1.0 (0–8)	2.5 (0–18)	0.025
Median number of 1-month VHDs, n (range)			1.0 (0–10)	5.0 (0–11)	0.004

CME = cystoid macular edema; OCT = optical coherence tomography; BCVA = best-corrected visual acuity; logMAR = the logarithm of the minimum angle of resolution; D = diopters; CRT = central subfield retinal thickness; VHD = vitreous hyper-reflective dot.

Results are expressed a mean ± standard deviation.

*Incidence of CME in total cases of each condition.

*P* values were calculated with the Fisher exact test for categorical variables and the Mann-Whitney U test for continuous variables.

**Table 3:** Multivariate logistic regression results for predicting CME development.

Factor	OR	95% CI	*P* value
Age, years	1.136	1.011 to 1.276	0.032
Sex, male	2.664	0.412 to 17.226	0.304
History of diabetes	0.174	0.010 to 3.142	0.236
Laterality, OD	0.495	0.068 to 3.595	0.487
Preoperative BCVA, logMAR	0.918	0.040 to 21.1651	0.957
Mean keratometric value, D	0.847	0.416 to 1.723	0.646
Anterior chamber depth, mm	3.185	0.181 to 55.992	0.428
Anterior chamber angle, degrees	0.958	0.789 to 1.163	0.665
Axial length, mm	1.562	0.515 to 4.735	0.431
Preoperative CRT, µm	0.995	0.952 to 1.041	0.836
Postoperative 1-week VHDs, n	1.934	1.154 to 3.242	0.012

CME = cystoid macular edema; OR = odds ratio, CI = confidence interval; BCVA = best-corrected visual acuity; logMAR = the logarithm of the minimum angle of resolution; D = diopters; CRT = central subfield retinal thickness; VHD = vitreous hyper-reflective dot.

**Table 4:** The correlation between the number of postoperative 1-week VHD and the development of CME.

	CME		
Number of VHDs	no	yes (%)	*P* value*
0	29	3 (9.4%)	0.001
1	18	0 (0%)	
2	6	2 (25.0%)	
3	4	1 (20.0%)	
4	2	1 (33.3%)	
5	1	0 (0%)	
6	1	1 (50%)	
8	1	1 (50%)	
18	0	1 (100%)	

VHD = vitreous hyper-reflective dot; CME = cystoid macular edema.

*chi-square test for trend (linear-by-linear association).

## Discussion

In the present study, we found that VHDs were frequently observed in OCT after uneventful phacoemulsification surgery. In addition, the number of VHDs detected at 1 week after the surgery was significantly associated with the development of CME at 1 month. The exact nature of VHDs in this study is a key feature that can explain the relation between VHDs and CME. However, it is not feasible to perform a pathologic analysis using a sample obtained from a functioning eye. Therefore, we estimated the etiology of VHDs based on a review of previous studies. The possible nature of VHDs includes, but not limited to, lens fragments, denatured proteins, and clumps of intraocular cells.

Ang et al. [16] reported that retrocapsular lens fragments are present in 17% of patients, suggesting the entry into the retrolenticular space via weak zonules. In the present study, VHDs may be retrocapsular lens fragments that sank into the posterior vitreous cavity over time. However, Liu et al. [17], [18] reported that all anterior vitreous specimens were negative for lens matter in a cytopathologic study of patients undergoing combined phacoemulsification and vitrectomy. Therefore, there is debate about the lenticular fragment access into vitreous cavity.

Yoshimoto et al. [19] demonstrated that vitreous collagen fibrils are condensed by laser-induced heat in bovine and pig eyes. The heat generated during phacoemulsification may denature vitreous fibrils and appear as VHDs in postoperative period.

Inflammatory cells usually infiltrate eyeball after surgery trauma. Saito et al. [12] previously reported that vitreous cavity cells could be visualized as hyper-reflective dots in OCT in uveitic eyes. However, it is noteworthy that their hyper-reflective dots ranged from 15 µm to 20 µm whereas the VHDs in our study were a least 20 µm in size in the definition. During phacoemulsification, zonular stress may cause epithelial cells to be released from the ciliary body. [20] These cells are another candidate for VHDs. Although the exact nature of VHDs is unclear, the significant association of VHDs with postoperative CME suggests that the VHD may be related to the postoperative inflammation and vascular permeability which are known as the most potent inducers of CME.

It is interesting that VHDs were also observed in the preoperative OCT. The nature of preoperative VHDs can be aggregation of collagen fibrils [21] or asteroid bodies presenting as hyper-reflective lesions in OCT. [22], [23] The vitreous body contains hyalocytes and fibroblasts, although the number is small. [24] Hyalocytes range from 10 to 15 µm in diameter, and fibroblasts are usually localized in the cortex near the ciliary processes, vitreous base, and adjacent to the optic disc. [24], [25] Therefore, these cells are not likely to be detected as VHD (vertical length of a least 20 µm) in our study.

The incidence of pseudophakic CME ranges from 4% to 11% in OCT after uneventful phacoemulsification cataract surgery, [6], [8], [26] and peaks at approximately 4 to 6 weeks. [2], [7] In the present study, the incidence of CME at 1 month after surgery was 13.9%. The incidence is slightly higher; however, it is difficult to compare due to different study designs, surgical techniques, subject characteristics, and definitions of CME. Currently in the literature, there is no validated or universally accepted method for reporting pseudophakic CME. [15], [27] Although fluorescein angiography remains the gold standard, [2] CME was defined based on OCT, but without reference to the vision loss in the present study. In addition, our definition of CME included not only the thickening of the retina, but also subtle intraretinal cystoid abnormalities without substantial increase in retinal thickness. It is known that intraretinal cystoid change without retinal thickening may be more common pattern in the macular changes which can be detected with OCT after cataract surgery. [15] With recent advance in technology, SD-OCT allows us to detect subtle changes of the macula. [28] Our definition of CME may result in not-significant difference in the mean postoperative BCVA between the groups with and without CME.

Using multivariate analyses, we found that the number of VHDs at 1 week after the surgery was significantly associated with the development of CME at 1 month. Therefore, VHDs observed at 1 week after surgery may be used as a risk factor (OR = 1.93) for CME development after routine cataract surgery. Intensive CME prevention using anti-inflammatory topical drugs may be helpful in the patients with high number of VHDs at 1 week after cataract surgery. It is noteworthy that the presence of VHDs at 1 week did not influence CRT and BCVA at 1 month in this study. These findings may be related to low incidence (17.5%) of CME in eyes with postoperative 1-week VHDs and the small difference of CRT between eyes with or without CME (mean CRT 296 µm vs. 251 µm, table 2). In addition, the same BCVA does not necessarily translate to identical functional vision. [29] For example, macular edema can affect lower more than higher contrast visual acuity. [30], [31] As expected, age was significantly associated with CME development in this study. Previous studies reported a positive correlation of CME with age, demonstrating an increased incidence of CME in older patients [3], [32].

This study has several limitations. Nuclear density and surgical techniques (phaco duration and power) may affect the presence of VHDs after surgery. However, retrospective study design prevented further analysis. Second, poor image quality due to cataract could interrupt the detection of VHDs in preoperative OCT. Even though we analyzed only SD-OCT with a signal to noise ratio of 0.6 or more, removal of the cataract will increase signal transmission of SD-OCT to the vitreous and may increase the detection of pre-existing VHDs. Another limitation of our study is that VHDs were manually counted. A VHD was at least 20 µm in size by definition in order to differentiate it from noise. However, ICCs for counting the number of VHDs were all >0.9. Another limitation is that VHDs were counted only in the posterior vitreous cavity near the retina. Therefore, the incidence of VHDs in the whole vitreous cavity was likely underestimated in the present study. If the source of VHDs were something migrating from the anterior part to the posterior part of the vitreous cavity after the surgery, it is unlikely in eyes with a longer axial length that VHDs would reach the posterior vitreous cavity and be detected with OCT. The finding that axial length of eyes with VHDs was significantly shorter, although the mean difference was measured only 0.6 mm, may be understood in the same context. In addition, high-definition 5-line raster scan images of the Cirrus™ HD-OCT were used to detect VHDs even though macular cube scan images show a more extensive region. The high-definition 5-line raster scan images with higher resolution allowed us to more reliably detect VHDs. Further study is needed to confirm whether images from other OCT devices can be used comparably to detect VHD. Another limitation relates to the lack of follow-up. Long-term prospective studies are needed to identify how long the VHDs remain and to understand the pathophysiology of VHDs.

In conclusion, the emergence of VHDs was frequently observed in OCT at 1 week and at 1 month after uneventful phacoemulsification surgery. The VHD observed at 1 week after the surgery was a risk factor for the CME development. To the best of our knowledge, this study is the first to report VHDs after phacoemulsification surgery and their clinical significance. Further studies to investigate the etiology of VHDs after cataract surgery are warranted.
